# *Ulva intestinalis* Extract Acts as Biostimulant and Modulates Metabolites and Hormone Balance in Basil (*Ocimum basilicum* L.) and Parsley (*Petroselinum crispum* L.)

**DOI:** 10.3390/plants10071391

**Published:** 2021-07-07

**Authors:** Roberta Paulert, Roberta Ascrizzi, Silvia Malatesta, Paolo Berni, Miguel Daniel Noseda, Mariana Mazetto de Carvalho, Ilaria Marchioni, Luisa Pistelli, Maria Eugênia Rabello Duarte, Lorenzo Mariotti, Laura Pistelli

**Affiliations:** 1 Department of Agronomic Sciences, Palotina Campus, Federal University of Paraná, 85.950-000 Palotina, Brazil; roberta@ufpr.br; 2 Department of Pharmacy, University of Pisa, 56126 Pisa, Italy; luisa.pistelli@unipi.it; 3 Department of Agriculture, Food and Environment, University of Pisa, 56124 Pisa, Italy; silvia061094@gmail.com (S.M.); paolo.berni@unipi.it (P.B.); ilaria.marchioni.16@gmail.com (I.M.); lorenzo.mariotti@unipi.it (L.M.); laura.pistelli@unipi.it (L.P.); 4 Department of Biochemistry and Molecular Biology, Federal University of Paraná, 81.531-980 Curitiba, Brazil; mdn@ufpr.br (M.D.N.); marianamazetto@ufpr.br (M.M.d.C.); nosedaeu@ufpr.br (M.E.R.D.); 5 Interdepartmental Research Center Nutraceuticals and Food for Health (NUTRAFOOD), University of Pisa, 56124 Pisa, Italy

**Keywords:** elicitor, macroalgae polysaccharide, essential oil, secondary metabolites, plant hormones, priming effect, seaweed extract

## Abstract

Natural elicitors from macroalgae may affect plant secondary metabolites. Ulvan is a sulfated heteropolysaccharide extracted from green seaweed, acting as both a plant biotic protecting agent, and a plant elicitor, leading to the synthesis of signal molecules. In this work, the aqueous extract of *Ulva intestinalis* L., mainly composed of ulvan, was used as foliar-spraying treatment and its eliciting effect was investigated in basil (*Ocimum basilicum* L.) and parsley (*Petroselinum crispum* L.). Antioxidant metabolites (polyphenols and carotenoids), volatile compounds (both in headspace emissions and hydrodistilled essential oils), and hormones (jasmonic acid, salicylic acid, salicylic acid 2-O-β-D-glucoside, abscisic acid, and azelaic acid) were quantified. The foliar-spraying treatment with *U. intestinalis* extract increased salicylic acid and its β-glucoside in parsley; in basil, it induced the accumulation of jasmonic and abscisic acids, indicating the presence of a priming effect. In basil, the elicitation caused a change of the essential oil (EO) chemotype from methyl eugenol/eugenol to *epi*-α-cadinol and increased sesquiterpenes. In parsley EO it caused a significant accumulation of 1,3,8-*p*-menthatriene, responsible of the typical “parsley-like” smell. In both species, the phenylpropanoids decreased in headspace and EO compositions, while the salicylic acid concentration increased; this could indicate a primarily defensive response to *U. intestinalis* extract. Due to the evidenced significant biological activity, *U. intestinalis* extract used as an elicitor may represent a suitable tool to obtain higher amounts of metabolites for optimizing plant flavor metabolites.

## 1. Introduction

Marine organisms can produce elicitors such as polysaccharides, and seaweeds represent a promising resource of bioactive substances [1]. For many years now, beneficial effects of spraying seaweed extracts on crop plants have been observed, and there is an increasing interest in oligo- and polysaccharides in plant immunity. Sulfated polysaccharides have gained attention in crop protection for their roles as priming agents, and elicitors that act as signaling molecules [2,3].

Ulvan is a high molecular weight sulfated polysaccharide found in the cell walls of *Ulva* species, mainly composed of rhamnose, xylose, uronic acids and sulfate groups [4]. For ulvan, different biological activities are reported, including immunomodulatory properties in fish, human and plant cells [5,6,7], as well as antioxidant ability [4]. These interesting features make ulvan a good candidate as a renewable compound for the development of nanoparticles [8] to be used for medical [5] and agricultural purposes [1,7]. As its oligomers are highly interesting as compounds with improved biological properties, ulvan lyases have been studied [9]. In terms of plant biological activity, *Medicago truncatula* plants sprayed with an extract of *Ulva* spp. (a mixture of several *Ulva* species) showed the induction of defense-related genes, with an almost complete protection against the pathogenic fungus *Colletotrichum trifolii* [10]. Furthermore, macroalgae extracts protected *Phaseolus vulgaris* L. against anthracnose [11], as well as grapevine, cucumber, common bean, barley and wheat against powdery mildew [12,13]. An ulvan-induced resistance was observed in *Arabidopsis thaliana* against black spot disease through increased activity of NADPH oxidase [14]. The elicitor effect of ulvan is demonstrated with the expression of systemic activity relaying on phenylalanine ammonia-lyase (PAL) and phenolic compounds accumulation in tomato seedlings [15]. The transcriptome was largely affected and the sulfated polysaccharide activated plant immunity through the jasmonic acid (JA) signaling pathway in *Medicago truncatula* and *Arabidopsis* hormone mutants [7]. These results highlighted the stimulation of plant defense responses by ulvan as a promising alternative strategy for crop protection. However, ulvan potential remains underestimated and, despite plant protection, to our knowledge, the effects of this polymer have not been tested in the secondary metabolism of medicinal and aromatic plants to produce flavorful molecules. Although the structure-activity relationship remains unknown, this is the first report based on *Ulva* extract stimulating responses in aromatic species.

To compare ulvan bioactivity to an agent whose use was already established, we have selected chitosan. Like ulvan, indeed, chitosan is a natural biopolymer which acts as a potential elicitor in agriculture [2,3], both enhancing the physiological responses and inducing the production of secondary metabolites [16].

Basil (*Ocimum basilicum* L.) and parsley (*Petroselinum crispum* L.) are largely cultivated crops, appreciated for their biological properties and taste. Their industrial cultivation is especially important in Mediterranean areas, but their consumption is widespread worldwide. Fresh or dried, their leaves have a long-established use as culinary herbs, flavoring ingredients or food garnishment. Their essential oils (EOs) are extracted from the leaves and used in cosmetic and pharmacological industries [17,18]. Their production is affected by some fungal diseases, and a new, green protecting agent could be useful to counteract such infections, which have caused production losses [19,20].

In this context, the present work investigated *Ulva intestinalis* aqueous extract (UIE) and chitosan foliar treatments on photosynthetic pigments, antioxidant molecules, production of volatile secondary metabolites emitted in the headspaces and composing the essential oils, and on plant hormone balance in basil and parsley plants. This should contribute to show evidence of a green sustainable biotechnological tool for optimizing plant flavor metabolites, as well as providing information on the priming and eliciting activity of UIE.

## 2. Results

### 2.1. Ulva intestinalis Extract (UIE) Analyses

The aqueous lyophilized extract isolated from *U. intestinalis* presented 44.6 (±1.7) and 14.3% (±1.7) of carbohydrate and sulfate groups, respectively. Uronic acids were detected (19.1% ± 2.1), corresponding to 43% of total carbohydrates. Proteins were also detected (11.3% ± 0.3). The monosaccharide composition showed rhamnose (65.2 mol%) and xylose (26.7 mol%), as major neutral sugars, with minor amounts of glucose and galactose. The presence of rhamnose, uronic acids and sulfate groups indicates that the polysaccharide ulvan is the main constituent of UIE. However, in the present study, *U. intestinalis* extract (UIE) was preferably used rather than ulvan in consideration of the protein content detected.

The elution profile of UIE obtained by HPSEC-RI-MALLS analysis is represented in Figure S1 (Supplementary Materials). The RI detector showed three different peaks. The peaks centered at 44 and 62 min were attributed to polymers of different molar masses while that at approximately 71 min indicates the presence of salts. Only the molecules eluted at 44 min were detected by the light scattering (MALLS) detector suggesting that this first peak is constituted by a high molar mass ulvan.

**Figure 1:**
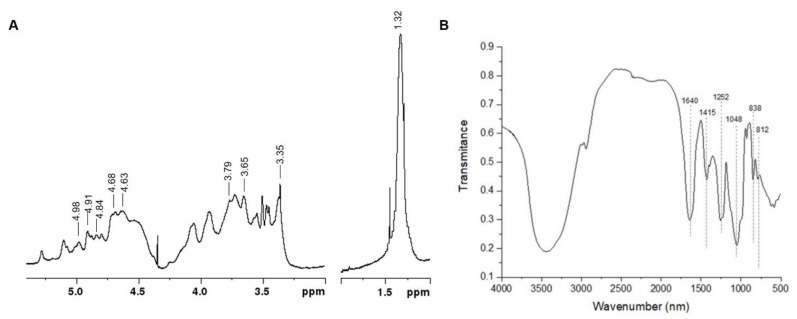
^1^H Nuclear magnetic resonance spectrum of *U. intestinalis* extract (UIE). Base frequency: 400.13 MHz. Solvent: D_2_O. Temperature: 70 °C. Spectrum calibrated with acetone (internal standard) at 2.225 ppm (**A**). Fourier-transform infrared spectrum of UIE (**B**).

The ^1^H NMR spectrum of UIE is presented in Figure 1A. In the anomeric region the resonance at 4.84 ppm was assigned to H-1 of α-L-rhamnose 3-sulfate (α-L-Rha 3S) linked to β-D-glucuronic acid (H-1 at 4.68 ppm), which correspond to ulvanobiuronic acid dyad (A_3s_) of ulvan. In the same region the signal at 4.91 ppm was attributed to H-1 of α-L-Rha 3S linked to β-D-xylose (H-1 at 4.68 ppm, U_3s_ ulvan dyad) and to β-D-xylose 2-sulfate (H-1 at 4.98 ppm, U_2s,3s_ ulvan dyad).

Characteristic sugar ring hydrogen resonances belonging to ulvans were also identified. The signals at 4.63 and 3.79 ppm correspond to H-3 and H-4 of α-L-Rha 3S, respectively. Moreover, the resonances at 3.65 and 3.35 ppm were attributed to H-4/H-3 and H-2 of glucuronic acid units, respectively. Finally, the intense resonance at 1.32 ppm was assigned to the methyl protons (H-6) of α-L-Rha, the main monosaccharide unit of ulvan. The NMR assignments are in accordance with previous studies of ulvans from different *Ulva* species [21,22].

The FTIR analysis of UIE showed a characteristic ulvan spectrum (Figure 1B), with strong absorbance bands at 1640, 1252, and 1048 cm^−1^ and small ones at 1415, 838, and 812 cm^−1^. The bands at 1640 and 1415 cm^−1^ were attributed to asymmetrical and symmetric stretching vibration of uronic acids carboxyl groups. The band at 1252 cm^−1^ was assigned to the presence of sulfate groups. The absorption band at 1048 cm^−1^ was attributed to C-O stretching of the main sugars, while the minor bands at 838 and 812 cm^−1^ are related with the sugar cycles. All these FTIR assignments are in agreement with those previously reported for ulvan by Robic et al. [23].

Therefore, chemical data and NMR and FTIR spectroscopic analyses indicate that UIE is mainly constituted by the sulfated heteropolysaccharide ulvan.

### 2.2. Elicitation of Leaves with UIE: Biochemical Analyses

Chlorophylls, carotenoids, total polyphenols and the radical scavenging activity were determined after foliar treatment with UIE, chitosan or water as control (Table 1). The analysis of photosynthetic pigments showed that parsley and basil were differently affected by UIE and chitosan treatments. Parsley showed similar amounts of chlorophyll a (Chla), chlorophyll b (Chlb), total chlorophyll (TChl), and carotenoids (TCar) in each trial, so the treatment with UIE or chitosan had no effect on its pigments content.

In basil, instead, significant different levels of TChl were observed in leaves treated with UIE in comparison with control ones, while the foliar application of chitosan did not result in significant changes. The discrepancy was attributed to the content of Chla and not to the Chlb. Leaves treated with water showed content of 973.37 (Chla) and 1222.23 (TChl) µg/g FW. In the chitosan sprayed leaves, the Chla and TChl contents were reduced to 898.2 and 1128.26 µg/g FW, while in the UIE-treated ones the amounts were 857.78 (Chla) and 1086.05 (TChl) µg/g FW. Basil leaves also showed a decrease in the TCar content when sprayed with UIE (219.86 µg/g FW) compared with control (248.37 µg/g FW), while chitosan did not induce a relevant difference compared with the control.

The total polyphenols content detected in both species did not exhibit statistical differences among the treatments and the control. However, the radical scavenging activity (IC_50_ of DPPH assay) was high in basil, with no differences among the treatments, while parsley leaves showed a lower scavenger activity (IC_50_ of DPPH assay).

### 2.3. Hormonal Profiles of Elicited Leaves

The endogenous hormonal levels in basil and parsley leaves were analyzed by GC-MS after treatment with chitosan or UIE in comparison with plants treated with water (Table 2). After treatment with UIE, endogenous levels of salicylic acid (SA) and salicylic acid β-glucoside (SAG) significantly increased in parsley and basil leaves. On the contrary, chitosan induced SA and SAG accumulation only in basil plants (Table 2). Basil and parsley showed a common increasing trend of abscisic acid (ABA) content, with the highest level in the treatment with UIE. However, jasmonic acid (JA) level accumulation was significantly different in the two plant species; UIE increased JA accumulation in basil while chitosan increased JA in parsley. Azelaic acid was detected only in parsley leaves, with its highest level in chitosan-treated plants; in basil it was below the instrument detection limit.

### 2.4. Volatiles Analyses: Headspaces (HSs) and Essential Oils (EOs)

#### 2.4.1. Basil

The complete compositions of the headspaces (HSs) and of the essential oils (EOs) are reported in Table S1 (Supplementary Materials). 1,8-Cineole, an oxygenated monoterpene, was detected as the most abundant compound in all the HSs, ranging from 44.74% in the chitosan-treated, 26.15% in UIE-treated and 18.63% in the control basil. Oxygenated monoterpenes were the most represented chemical class of compounds in both the treated samples, in which linalool showed relative abundances over 4.5 times induced by UIE. The control HS, instead, was mainly composed of sesquiterpene hydrocarbons (more than 39%). Of this latter class, (*E*)-β-farnesene and *trans*-α-bergamotene were the most abundant in all the samples, thus showing quantitative rather than qualitative differences among the treatments. Phenylpropanoids also showed quantitative differences induced by the elicitors, which caused a decrement in both eugenol and methyl eugenol in the HSs. Monoterpene hydrocarbons, instead, did not exhibit major quantitative differences caused by the treatments.

Phenylpropanoids constituted over 65% of the total EO composition of the control sample, which showed a methyl eugenol/eugenol chemotype. Both the elicitors significantly reduced this chemical class, as evidenced in the HSs. Sesquiterpenes (hydrocarbon and oxygenated forms) were significantly accumulated by both chitosan and UIE. Sesquiterpene hydrocarbons were the most abundant compounds in both the treatments; among them, *trans*-α-bergamotene, (*E*)-β-farnesene and germacrene D were the most represented. As was found for the HSs, the change induced in this chemical class was quantitative rather than qualitative; moreover, the same was true for oxygenated sesquiterpenes, whose most represented compound was *epi*-α-cadinol in all the EO compositions. Interestingly, both elicitors caused a change in basil chemotype, which switched to *trans*-α-bergamotene and *epi*-α-cadinol in response to the chitosan and UIE treatments, respectively. A reduction of the oxygenated monoterpenes was observed in both treatments.

**Figure 2:**
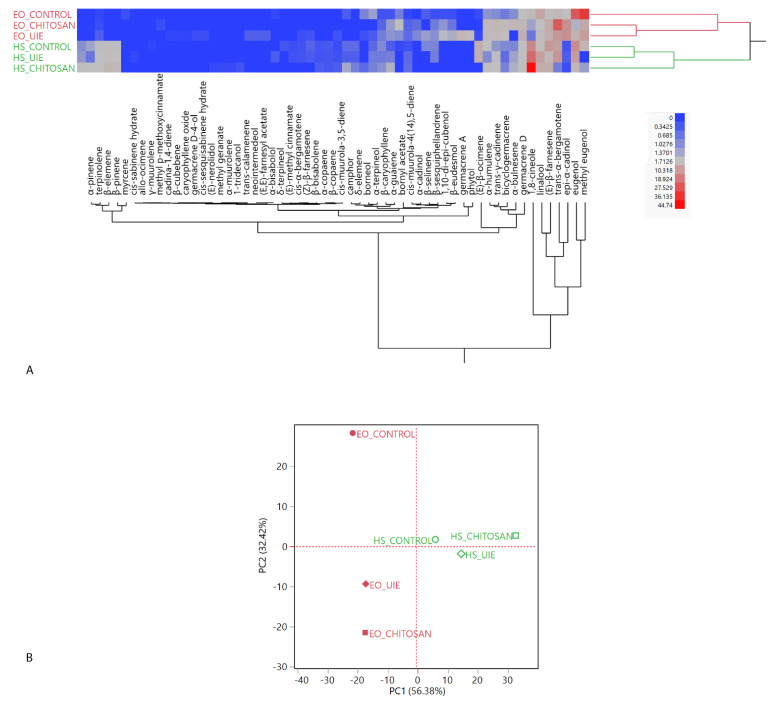
Two-way dendrogram of the hierarchical cluster analysis (**A**) and score plot of the principal component analysis (**B**) performed on the complete compositions of basil (*Ocimum basilicum* L.) leaf headspaces (HS) and essential oils (EO). The plants were treated by foliar spraying with UIE, chitosan or water as control.

Figure 2A illustrates the two-way dendrogram of the hierarchical cluster analysis (HCA), where, among the EOs (red macro-cluster), the highest proximity was revealed between the two treated samples. Headspaces grouped in the green macro-cluster, instead, showed a higher similarity among the control and the UIE-treated samples. The majority of the dissimilarity among the treatments was due to the most represented compounds common to all the EOs and HSs (eugenol, methyl eugenol, *epi*-α-cadinol, *trans*-α-bergamotene, (*E*)-β-farnesene, linalool, and 1,8-cineole) vs. all the remaining compounds, which were less abundant in all the compositions.

The score plot of the principal component analysis (PCA) is depicted in Figure 2B; the loadings plot is reported in Figure S2 (Supplementary Materials). The higher similarity level in the composition of the essential oils of the two treated basil samples was evident in the PCA score plot, as well: the control basil EO was plotted by itself in the upper left quadrant (PC1 < 0, PC2 > 0), while the two treated ones were positioned in the bottom left quadrant (PC1 and PC2 < 0).

#### 2.4.2. Parsley

The complete compositions of the headspaces (HSs) and of the essential oils (EOs) are reported in Table S2 (Supplementary Materials). Headspaces were all mostly composed of monoterpene hydrocarbons. Among these compounds, β-phellandrene was the most represented in the control sample, whilst in both the treated samples it was 1,3,8-*p*-menthatriene. The latter is reported as a characteristic aroma-active compound in this species, and it is described as having a “parsley-like” smell [24]. Phenylpropanoids followed, showing a significant reduction in the UIE-treated leaves. Apiole was identified with the highest relative abundance in the control and chitosan-elicitated samples and myristicin was, instead, found as the most relevant phenylpropanoid in the UIE-treated one. The third detected chemical class in terms of relative concentration was the sesquiterpene hydrocarbons one, which exhibited a significant accumulation in samples treated with both elicitors, especially the chitosan-treated one. Germacrene D, in particular, was significantly increased, while β-sesquiphellandrene showed the opposite trend.

**Figure 3:**
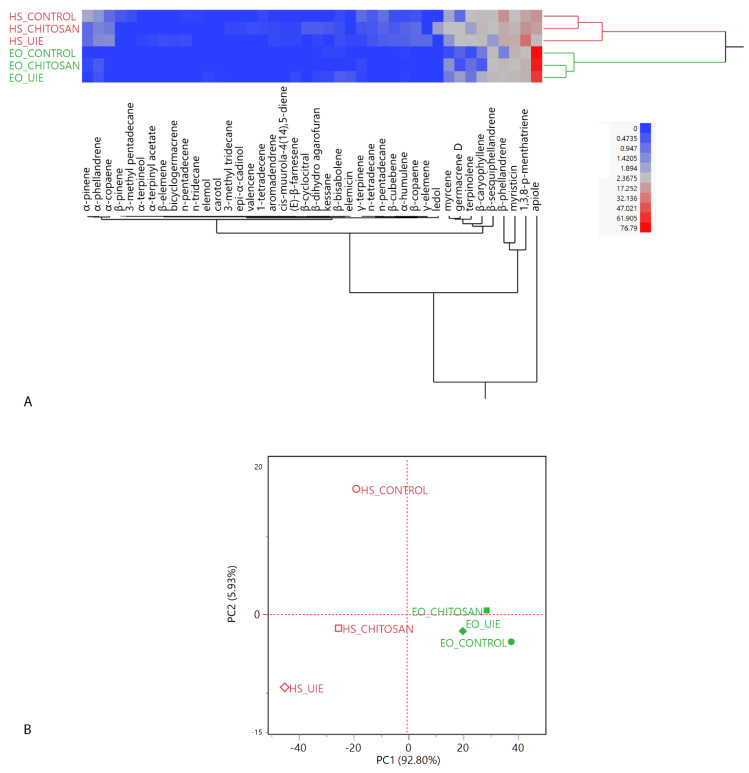
Two-way dendrogram of the hierarchical cluster analysis (**A**) and score plot of the principal component analysis (**B**) performed on the complete compositions of parsley (*Petroselinum crispum* L.) leaf headspaces (HS) and essential oils (EO). The plants were treated by foliar spraying with UIE, chitosan or water as control.

As emerged from the EO compositions, all parsley samples exhibited an apiole chemotype and it represented over 60% of all the EOs, with its lowest relative abundance (61.27%) in the UIE-treated leaves. Both elicitors reduced the phenylpropanoid fraction of EOs, which was more pronounced in the UIE-treated specimen. Monoterpene hydrocarbons, instead, were detected at statistically significant higher relative abundances in both the elicitated samples: the most quantitatively relevant compounds of this class were 1,3,8-*p*-menthatriene and β-phellandrene. Sesquiterpene hydrocarbons, mainly represented by β-sesquiphellandrene, instead, exhibited a different trend depending on the used elicitor: chitosan reduced their accumulation, while UIE increased it. The two-way dendrogram of the hierarchical cluster analysis (HCA) is reported in Figure 3A. While the HS macro-cluster (red) showed a higher proximity of the chitosan-treated sample headspace with the control, compared with the UIE one, both the elicitors induced higher differences in the EO composition (green macro-cluster), in which the control sample is sub-clustered by itself. The majority of the dissimilarity among the samples was due to apiole and 1,3,8-*p*-menthatriene presence.

The loading plot is reported in Figure S3 (Supplementary Materials) and the score plot of the principal component analysis (PCA) is shown in Figure 3B. The PCA evidenced an overall more pronounced influence on parsley HS compositions, compared with the EOs: the former samples are, indeed, plotted in a more scattered fashion on the score plot. All the headspaces are plotted in the left quadrants (PC1 < 0), while the EOs are all positioned in the right ones (PC1 > 0). The control sample headspace is plotted in the upper left quadrant (PC1 < 0, PC2 > 0) due to its β-phellandrene content, whose vector points towards that quadrant (Figure S6 of the Supplementary Materials). The other headspaces are both plotted on the bottom left quadrant (PC1 and PC2 < 0): this positioning is mostly due to their 1,3,8-*p*-menthatriene vector. The UIE-treated sample, though, also exhibited a higher myristicin relative content. For the EOs samples, instead, given their high relative concentrations of apiole (all over 70%), the grouping is closer on the score plot. The control and UIE-treated sample EOs, however, are plotted in the bottom right quadrant, slightly apart from the chitosan-treated sample: this was probably due to their higher myristicin relative content (Figure S3 of the Supplementary Materials).

## 3. Discussion

Elicitors might represent a bioengineering technique for optimizing plant flavor metabolites, particularly targeted to cyber-agriculture systems, where the environmental conditions are artificially controlled [25]. The accumulation of bioactive compounds in plant tissues can be induced by the exogenous application of natural elicitors. In the last decades, the use of green seaweeds of *Ulva* genus has gained interest because they are abundant, and their polysaccharides are promising bioactive compounds for plant disease protection of potentially relevant commercial use. In particular, ulvan is a sulfated polymer with a range of desirable biological properties [4], such its eliciting activity, since it is non-toxic, biodegradable, and food-grade.

Ulvan usually has large molar mass, a degree of sulfation up to 35% and is typically composed by rhamnose, xylose, and uronic acids (glucuronic and iduronic acids) [4]. Other monosaccharides are often described as constituents (e.g., glucose, galactose, arabinose and mannose); however, their presence as components of ulvan is not clear and the polysaccharide chemical composition can vary based on the algae species, eco-physiology and extraction methods [4]. In our work, the analyses performed on *U. intestinalis* extract (UIE) confirmed that the aqueous extract is mainly composed by the sulfated polysaccharide ulvan.

Ulvan is able to reduce the disease severity in different host-pathogen biosystems [7], by stimulation of specific enzymatic activities, salicylic acid production, and increase in phenolic compounds [15,26], as well as being involved in the jasmonic acid signaling pathway in leaves of *Arabidopsis thaliana* (L.) [7]. Ulvan induced an activation of the plant immune system through the expression of the defense-related marker gene *PR10* up to 15 days in *Medicago* young plants [10]. It was also reported that rice and wheat cultured-cells were strongly primed by the pre-treatment with ulvan, increasing the oxidative burst triggered by chitin hexamer or chitosan [13]. Ulvan bioactivity is, thus, multi-faceted, as it prepares the plant to better respond to stress (priming effect), it acts on plant genes involved in the immune response, as well as on enzymatic activities in the biosynthetic pathways of stress-protective metabolites. On the basis of the induced resistance against pathogenic fungi, recently, the ulvan seaweed extract has been selected as new sustainable and highly viable strategy for the stimulation of plant immunity [26].

Nothing is known about ulvan effect in aromatic and medicinal plants, that are used and consumed for their biological properties and the aroma of their volatile compounds. Here, for the first time, we showed that an aqueous extract of *U. intestinalis*, mainly composed of ulvan (UIE), can modulate hormone balance and aroma profile in parsley and basil, important aromatic plants.

According to the plant hormones determination, the UIE was able to accumulate higher amounts of SA, SAG, and ABA. On the other hand, JA increased in basil, together with SA, SAG and ABA. SA and JA participate in providing plant immunity to various stresses [27,28]. Generally, SA-dependent defense signaling is known to be antagonistic to the JA-dependent one, and the induction of one pathway strongly suppresses the other [28]. However, SA and JA hormones are not exclusively antagonistic. Their mutual behavior needs to be carefully analyzed in different plant-pathogen systems [29]. In this work, the distinctive presence of JA and SA in parsley and basil can be attributed to the different behavior of these plant species; it is known that parsley cell cultures triggered several defense responses after treatment with SA [30]. Interestingly, the SAG was highly accumulated in parsley and basil after *U. intestinalis* extract treatment (Table 2). The increment of this metabolite is important during the pre-challenge priming stage, because it can be rapidly converted into its active form once the interaction with the pathogen has been established [31]. El Modafar et al. [15] demonstrated that ulvans and oligo-ulvans expressed an elicitor effect increasing phenylalanine ammonia-lyase (PAL) activity followed by an accumulation of phenolic compounds in tomato leaves. As the PAL enzyme is also involved in SA biosynthesis, the results of this study might indicate that basil and parsley response to the elicitors is primarily defensive, since PAL metabolic pathway is mainly addressed towards the production of SA and SAG, both increased by the elicitation treatments. The leaves of parsley and basil showed an increase in ABA content, after spray-treatment with both chitosan and UIE. The correlation between ABA content and SA for the closure of stomata has been demonstrated [32]. However, in basil, the ABA content could be correlated to the reduced content of photosynthetic pigments (chlorophyll a and carotenoids). The ABA level was high in both plants treated with UIE. Under certain abiotic stress conditions, such as freezing and salt stress, ABA and SA together seem to positively regulate stress tolerance response [33]. The low chitosan concentration (0.1 g mL^−1^) applied in the present work increased ABA and JA level in parsley, while with SA, SAG in basil, although this was not sufficient to induce significant changes in antioxidant compounds, as observed in other studies on beans and pepper [34,35]. In the case of parsley treated with chitosan, the JA accumulation is accompanied by that of AZA, which was not detected in basil (Figure 3). AZA is a natural inducer of plant defenses, as it is considered a mobile metabolite involved in priming defenses [36] and in long-distance SAR signaling. In brief, the UIE treatment stimulated SA biosynthesis in basil and parsley, while chitosan enhanced SA biosynthesis in the former and JA in the latter.

Chitosan is known to act as antimicrobial agent [2] and to confer resistance against several abiotic stresses, such as water deficit, salinity, heat stress and heavy metal toxicity [16]. In basil, foliar application of chitosan increased plant growth under drought and showed the capacity to scavenge ROS [16]. In cowpeas, chitosan alleviated the effect of drought by enhancing chlorophylls and total carbohydrates [37]. However, the very low concentration of chitosan used in our work (100 μg/mL), in comparison with those reported in the literature, suggests that chitosan had no effect on parleys leaves, neither on the chlorophyll system nor the antioxidant system. In apple fruits treated with ulvan, Abouraïcha et al. [27] reported the activation of antioxidant-related enzymes and the synthesis of lignin and phenolic compounds, considered a priming effect to induce the natural defense and protection against fungi. However, the foliar treatment with UIE did not increase such properties in the specimens of basil and parsley studied in the present work (Table 1), suggesting that, in these aromatic plants, the elicitation effect of UIE is sufficient to stimulate a very early priming defense, without a significant difference in polyphenols and antioxidant activity. On the other hand, the phenylpropanoids biosynthesis appeared to be decremented.

In the present study, indeed, the elicitation of basil and parsley with both treatments reduced the phenylpropanoid content in the EOs of the treated samples. This finding is in contrast with several published studies, where chitosan is reported as an enhancer of the phenylalanine ammonia lyase and of the chavicol-O-methyl transferase enzymes, involved in the phenylpropanoid biosynthesis [38,39,40]. The different response of the two species to the elicitation treatments was also evident on the different level of compositional dissimilarity induced in the HS and the EO compositions. In basil, both elicitors induced more evident differences on the EO compositions, while the HSs of parsley were more influenced by the elicitation treatments compared with EOs. This might be explained as a faster environmental response of parsley compared with basil; the latter, however, reacts by changing its chemotype. Basil shows numerous chemotypes, with cultivars and geographical origin being the main reasons for such variability in its essential oil composition. Here, the influence of chitosan and UIE on the basil chemotype induced an evident change. Parsley chemotype, on the contrary, was not influenced, as all its EO samples exhibited apiole as the most abundant compound.

## 4. Materials and Methods

### 4.1. Algal Material

Green seaweed was collected at Spezzina Itticoltura, Le Grazie (Italy) (N 44.0650708, E 9.8285929,1) on January 2019. The fresh material was identified in the Biology Department of Pisa University as *Ulva intestinalis* Linnaeus (formerly *Enteromorpha intestinalis*). After washing with tap water, the algae were air-dried, grounded into a fine powder before extraction.

### 4.2. Preparation of Ulva Intestinalis Extract (UIE)

To obtain UIE, fresh algae (184 g fresh weight) were autoclaved in 1 L of distilled water at 90 °C for 2 h to ensure the absence of microorganisms. The aqueous solution was separated from the algal residues by filtration through a nylon mesh and concentrated under rotavaporator model R-114 (Buchi Labortechnik AG, Flawill, Switzerland) to about 200–300 mL. Soluble compounds were precipitated with the addition of 2.5 volumes of ethanol for 48 h at −20 °C [10]. The precipitated was recovered and lyophilized, giving rise to UIE (1.79 g).

### 4.3. UIE Composition Analyses

The total carbohydrate content was estimated using rhamnose as standard (10–50 μg) and spectrophotometrically read at 480 nm [41]. The uronic acids content was determined with glucuronic acid as standard (10–40 μg) and spectrophotometrically read at 525 nm [42]. The protein content was measured at 750 nm using the Folin-Ciocalteau reagent (Merck, KGaA, Darmstadt, Germany) with bovine serum albumin (5–100 μg) as the standard [43]. Sulfate content was measured using sodium sulfate as standard (20–200 μg) and spectrophotometrically read at 360 nm [44]. The spectrocolorimetric analyses (SpectraMax 340, Molecular Devices, San Jose, CA, USA) were performed in triplicate and the results were shown in mean ± SD (Standard Deviation).

The UIE sample was hydrolyzed (1 mol/L TFA, 100 °C, 4 h), reduced (NaBH_4_, 16 h, 25 °C), and acetylated (acetic anhydride 0.5 mL and sodium acetate as catalyst, 1 h, 100 °C). The alditol acetates derivatives were analyzed by gas chromatography with a Thermo GC Trace Ultra Fit chromatograph (Thermo Fisher Scientific, Waltham, MA, USA) equipped with a flame-ionization detector (FID). The capillary column (30 m × 0.25 mm i.d.) was coated with DB-225 (Agilent Technologies Inc., Santa Clara, CA, USA). Chromatography was run isothermally at 210 °C and the injector and FID temperatures were set at 250 °C. Nitrogen was used as carrier gas (1 mL/min). The alditol acetates derivatives were identified by their GC retention times compared with those of authentic analytical standards purchased from Merck (Darmstadt, Germany).

### 4.4. High-Pressure Size-Exclusion Chromatography (HPSEC) Coupled to Refractive Index (RI) and Multi-Angle Laser Light Scattering (MALLS) Detectors Analysis of UIE

The HPSEC-RI-MALLS analysis was carried out at 25 °C on UIE (1 mg/mL) using a multi-detection equipment with a Waters2410 RI detector and a Wyatt Technology Dawn-F MALLS detector (Wyatt Technology, Santa Barbara, CA, USA). Four columns (Ultrahydrogel 2000, 500, 250, and 120) were connected in series (Waters Corporation, Milford, MA, USA) and coupled with the multi-detection equipment. The chromatographic system total volume is equivalent to 71 min. The eluent was NaNO_2_ 0.1 mol/L containing NaN_3_ (0.2 g/L). The obtained HPSEC data were collected and analyzed with ASTRA software (Wyatt Technology, Santa Barbara, CA, USA).

### 4.5. NMR and FTIR Analyses of UIE

The ^1^H NMR spectrum was recorded using a Bruker Advance DRX400 NMR spectrometer (Bruker, Billerica, MA, USA) at 70 °C. A 5 mm multinuclear inverse detection probe was used at a base frequency of 400.13 MHz. The *U. intestinalis* extract sample was dissolved in D_2_O (99.9%) at 20 mg/mL and deuterium exchanged (3×). The acquisition parameters were previously reported [45]. Chemical shifts were expressed relative to acetone (internal standard) at 2.225 ppm.

Fourier-transform Infrared (FTIR) analysis was performed using a Bio-Rad spectrometer (model FTS, Hercules, CA, USA) using KBr discs (UIE at 1% *w*/*w*). A total of 64 scans were performed at a resolution of 2 cm^−1^ and wavenumber range from 4000 to 400 cm^−1^.

### 4.6. Plant Material and Treatment

Basil (*Ocimum basilicum* L.) and parsley (*Petroselinum crispum* L.) plants were obtained from Azienda Agricola L’Ortofruttifero (Pisa, Italy). They were selected as target species as they are widely cultivated in the Mediterranean area, and are considered as model plants for different studies, either for the exposition to pathogen (fungi) attacks or other abiotic stresses.

Three plants per pot were grown in a Brill Orto-pack Bio (MT) organic substrate consisting of blonde peat (fraction 0–5), coir (light fraction) and black peat (fraction 0–6) with a bulk density of 270–320 g/L, an air volume of 20/25% and a water retention capacity of 5.8 g/g. The substrate was characterized as follows: pH 5.5–6.5; EC <1 mS/cm; N 365 mg/L; P 125 mg/L; K 167.5 mg/L; Mg 12 mg/L; Fe 15 mg/L; S 38 mg/L. Irrigation took place daily by distilled water sprinkling without the use of fertilizers or nutritional solutions. The plants were grown for one month in the greenhouse at 25 °C during the day and 20 °C at night with a photoperiod of 16 h: 8 h (day: night) before the elicitation. Chitosan polymer was used as general elicitor of plants and purchased from Sigma-Aldrich Inc., St. Louis, MO, USA. The polymer was dissolved with 0.02% of acetic acid and used at a final concentration of 100 μg/mL for plant treatment. The chitosan concentration used was based on the previous study by Vander et al. [46]. Aqueous solutions were sprayed on the aerial part of plants until run-off point (4 mL per plant), and two consecutive treatments were performed. *U. intestinalis* extract (UIE, 1 mg/mL in distilled water), chitosan (100 μg/mL) or water were sprayed onto leaves surface of 1-month-old plants. For each treatment, 12 plants were used. The second foliar treatment was done 1 day after the first treatment. Two days after the second treatment, fresh leaves were homogeneously harvested and used for headspace analyses and essential oil hydrodistillation, and stored at −80 °C, for biochemical and hormonal analyses.

### 4.7. Biochemical Analyses of Leaves

Chlorophylls (a, b, total) and carotenoids were extracted from 100 mg fresh leaves with 5 mL of methanol 99% and incubated for 24 h at 4 °C, centrifuged and quantified in a UV-1800 spectrophotometer (Shimadzu Corporation, Kyoto, Japan) at 665, 652 and 470 nm [47]. Total soluble polyphenolic compounds and the free radical scavenging activity (DPPH assay) were determined as already described [48] using 100 mg fresh plant samples. Total soluble polyphenolic compounds were referred as gallic acid as standard [48] and the antioxidant activity was expressed as IC_50_, the concentration (μg/mL) of the extract providing 50% of antioxidant activity. The presented data are the mean of three independent replications.

### 4.8. Hormonal Level Quantification

Briefly, 500 mg of leaves (fully developed, pool of at least three different and randomly collected) were homogenized in cold 80% (*v*/*v*) methanol (1:5, *w*/*v*). Internal standards [^2^H_6_]-ABA (OlChemlm Ltd., Olomouc, Czech Republic), [^2^H_4_]-SA and [^2^H_5_]-JA (CDN Isotopes Inc., Quebec, Canada) were added. Methanol was evaporated under a vacuum at 35 °C, adjusted to the pH to 2.8, thus the aqueous phase was partitioned against ethyl acetate. Organic phases were collected and stored at 4 °C in darkness until HPLC analysis. Jasmonic acid (JA), salicylic acid (SA), salicylic acid 2-O-β-D-glucoside (SAG), abscisic acid (ABA), and azelaic acid (AZA) were separated by reversed phase HPLC [49].

Fractions corresponding to the elution volumes of standard of SA and AA were collected, dried and silylated with N,O-bis(trimethylsilyl)trifluoroacetamide containing 1% trimethylchlorosilane (Pierce, Rockford, IL, USA) at 70 °C for 1 h. Fractions corresponding to the elution volumes of standard of JA and ABA were collected, dried and methylated with diazometane. The GC-MS analysis was performed as previously reported [49]. Plant hormones were identified by comparing full mass spectra with standard compounds. The concentration of each plant hormone in the extracts was determined from the peak area ratio of labelled and non-labelled ions of internal standard and endogenous hormone, respectively. Final data were means of three biological replicates.

### 4.9. Headspace Solid Phase Micro-Extraction (HS-SPME)

The headspace spontaneous volatile emissions of treated basil and parsley samples were compared with their untreated control counterparts. Triplicates were performed for each analysis. For each replica, six leaves from six different plants were added in a glass vial closed with aluminum foil. As reported in Giuliani et al. [50], the equilibration was performed at room temperature for 30 min before sampling. A Solid Phase Micro-Extraction (SPME) device (Supelco, St. Louis, MO, USA) coated with polydimethylsiloxane (PDMS, 100 μm) was used for sampling, which was accomplished in an air-conditioned room (22 ± 1 °C) to guarantee a stable temperature. A 3 min sampling interval was experimentally determined to ensure the optimal fiber adsorption of the volatiles, avoiding both over- and under-saturation of the fiber. Once sampling was finished, the fiber was withdrawn into the needle and transferred to the injection port of the GC-MS system. Blanks were performed before each first SPME extraction, and randomly repeated during each series. Quantitative comparisons of relative peaks areas were performed between the same compounds in different samples.

### 4.10. Essential oil (EO) Hydrodistillations

For all the samples, 50 g of fresh leaves from six different plants were cut and subjected to hydrodistillation in a standard Clevenger apparatus for 2 h. Each extraction was performed in triplicate. Immediately after each distillation, 1 μL of essential oil was injected in the GC-MS apparatus after dilution in *n*-hexane HPLC grade at 5%.

### 4.11. Gas Chromatography-Electron Impact Mass Spectrometry (GC–EIMS) Analyses and Peak Identifications

Gas chromatography–electron impact mass spectrometry (GC–EIMS) analyses were performed with an Agilent 7890B gas chromatograph (Agilent Technologies Inc., Santa Clara, CA, USA) equipped with an Agilent HB-5MS (Agilent Technologies Inc., Santa Clara, CA, USA) capillary column (30 m × 0.25 mm; coating thickness 0.25 μm) and an Agilent 5977B single quadrupole mass detector (Agilent Technologies Inc., Santa Clara, CA, USA). Analytical conditions were as follows: injector and transfer line temperatures were set to 220 and 240 °C, respectively; the oven temperature was programmed to rise from 60 to 240 °C at 3 °C/min; helium was used as carrier gas, with a 1 mL/min flow; 1 μL of 0.5% HPLC grade *n*-hexane solution was injected; the split ratio was 1:25. The acquisition was perfomed in full scan, within a 30–300 *m*/*z* range, with a scan time of 1.0 s. The identification of the constituents was based on the comparison of their retention times with those of authentic samples, comparing their linear retention indices relative to the series of *n*-hydrocarbons. Computer matching was also used against commercial [51], our laboratory-developed mass spectra library, built up from pure substances and components of commercial essential oils of known composition, and MS literature data [52].

### 4.12. Statistical Analyses

Biochemical data were statistically analyzed by one-way ANOVA followed by Fisher’s probable least-squares difference test with cut-off significance at *p* ≤ 0.05. Hormonal data were analyzed by one-way and 2way ANOVA followed by Tukey’s test (StatView^®^, Version 5.0, SAS^®^ Institute Corporation, Cary, NC, USA).

The multivariate statistical analyses of the headspace and essential oil compositions, as well as the ANOVA on the HS and EO compositions were performed with the JMP software package (SAS Institute, Cary, NC, USA). The hierarchical cluster analyses on both species were carried out using Ward’s algorithm with Euclidean distances on normalized, unscaled data. The principal component analyses (PCA) were performed selecting the two highest principal components (PCs) obtained by the linear regressions operated on mean-centered, unscaled data [53]. For the principal component analysis of the basil samples, the covariance data matrix was a 58 × 6 matrix (57 individual compounds × 6 samples = 348 data), with a total studied variance of 88.80% (of which 56.38% on PC1 and 32.42% on PC2). For the principal component analysis of the parsley samples, the covariance data matrix was a 56 × 6 matrix (56 individual compounds × 6 samples = 336 data), with a total studied variance of 98.73% (of which 92.80% on PC1 and 5.93% on PC2).

## 5. Conclusions

The success of culinary herbs among consumers is due to their essential oil composition, since it confers them the expected flavor and aroma. The influence of elicitors on herb essential oil composition, which was particularly evident in basil, might be used as a method of obtaining different volatile compounds or to induce the presence of desirable compounds, depending on the consumers-driven preference. The *Ulva intestinalis* extract (UIE) might, indeed, represent a renewable and non-toxic resource, able to modify plant metabolic pathways for optimizing plant flavor metabolites. As UIE can regulate signaling pathways in basil and parsley, it could be used as an environmentally-friendly strategy for optimizing plant flavor metabolites without genetic modification involved. Further studies are necessary to evaluate the correct UIE amount necessary to improve the essential oil composition and the tolerance to different stress conditions.

## Figures and Tables

**Table 1:** Determination of foliar pigments (chlorophyll a, b and total; expressed as µg/g FW), carotenoids (µg/g FW), total polyphenols content (mg GAE/g FW), IC_50_ of the free radical (DPPH) scavenging activity (IC_50_ µg/mL) of one-month-old basil and parsley plants sprayed with UIE, chitosan or water as control (concentration used in materials and methods). Data are expressed as means ± SD (*n* = 3) and were statistically analyzed by one-way ANOVA followed by Fisher’s probable least-squares difference test: different lowercase letters (a,b) indicate significant differences (*p* < 0.05) among the samples along the same line.

	Basil			Parsley		
	Water	Chitosan 100 μg/mL	UIE 1 mg/mL	Water	Chitosan 100 μg/mL	UIE 1 mg/mL
Chlorophyll a (Chla) µg/g FW	973.37 ± 23.55 a	898.2 ± 21.74 ab	857.78 ± 16.71 b	1112.64 ± 247.03 a	1424.95 ± 26.83 a	1294.21 ± 140.59 a
Chlorophyll b (Chlb) µg/g FW	248.86 ± 6.41 a	229.94 ± 5.56 a	228.27 ± 2.68 a	358.75 ± 66.20 a	433.26 ± 8.97 a	398.15 ± 41.08 a
Total Chlorophylls (TChl) µg/g FW	1222.23 ± 29.92 a	1128.26 ± 27.30 ab	1086.05 ± 19.28 b	1471.39 ± 312.97 a	1858.21 ± 35.14 a	1692.35 ± 181.66 a
Ratio Chl a/Chl b	3.91 a	3.91 a	3.76 b	3.06 a	3.27 a	3.25 a
Total Carotenoids (TCar) µg/g FW	248.37 ± 5.46 a	230.1 ± 6.41 ab	219.86 ± 4.67 b	269.71 ± 63.79 a	342.4 ± 18.13 a	337.22 ± 32.57 a
Ratio TCar/TChl	0.20 a	0.20 a	0.20a	0.18 a	0.18 a	0.20 a
Total polyphenols (TP) mg GAE/g FW	2.63 ± 0.25 a	2.67 ± 0.14 a	2.61 ± 0.79 a	4.52 ± 0.34 a	5.52 ± 0.24 a	5.49 ± 0.26 a
DPPH assay (IC_50_ µg/mL)	1.56 ± 0.29 a	1.24 ± 0.04 a	1.13 ± 0.14 a	10.78± 1.74 a	6.88 ± 0.41 a	8.47 ± 0.99 a

**Table 2:** Endogenous levels (ng/g FW) of salicylic acid (SA), salicylic acid 2-O-β-D-glucoside (SAG), jasmonic acid (JA), abscisic acid (ABA) and azelaic acid (AZA) in basil and parsley leaves treated with UIE, chitosan or water as control. Data are expressed as means ± SD (*n* = 3) and were statistically analyzed by one-way ANOVA followed by Tukey’s HSD test: different lowercase letters (a,b) indicate significant differences (*p* < 0.05) among the samples along the same line.

	Basil			Parsley		
	Water	Chitosan 100 μg/mL	UIE 1 mg/mL	Water	Chitosan 100 μg/mL	UIE 1 mg/mL
Salicylic Acid (SA) ng/g FW	45.30± 2.68 c	253.04 ± 14.72 a	149.68 ± 17.72 b	225.06 ± 8.40 b	204.80 ± 5.50 b	977.98 ± 12.19 a
Salicylic Acid Glucoside (SAG) ng/g FW	1.39 ± 0.18 c	33.81 ± 2.90 b	236.19 ± 6.93 a	113.71 ± 2.71 b	123.72 ± 8.21 b	1643.57 ± 42.23 a
Jasmonic Acid (JA) ng/g FW	182.39 ± 5.83 b	135.27 ± 12.12 c	350.58 ± 18.12 a	84.06 ± 3.75 b	302.31 ± 2.89 a	65.87 ± 3.37 c
Abscisic Acid (ABA) ng/g FW	71.28 ± 1.36 b	89.66 ± 2.37 b	135.54 ± 15.07 a	62.38 ± 4.18 c	93.81 ± 9.35 b	152.42 ± 3.47 a
Azelaic Acid (AZA) ng/g FW				77.19 ± 4.00 b	101.30 ± 10.19 a	73.49 ± 5.25 b

## Data Availability

The data reported in this work are new and original, and they are fully reported in the present manuscript and its Supplementary Materials.

## Supplementary Materials

The following are available online at https://www.mdpi.com/article/10.3390/plants10071391/s1, Figure S1: Elution profile of *U. intestinalis* extract (UIE) as determined by high-pressure size-exclusion chromatography (HPSEC) coupled to refractive index (RI), and multi-angle laser light scattering (MALLS) detectors; Figure S2: Loadings plot of the principal component analysis (PCA) performed on the complete compositions of basil leaf headspaces (HS) and essential oils (EO); Figure S3: Loadings plot of the principal component analysis (PCA) performed on the complete compositions of parsley leaf headspaces (HS) and essential oils (EO); Table S1: Complete compositions of the headspaces and essential oils of basil (*Ocimum basilicum* L.) leaves treated by foliar spraying with *Ulva intestinalis* extract, chitosan or water (control); Table S2: Complete compositions of the headspaces and essential oils of parsley (*Petroselinum crispum* L.) leaves treated by foliar spraying with UIE, chitosan or water (control).
