# Supplementary material for: Ulva intestinalis Extract Acts as Biostimulant and Modulates Metabolites and Hormone Balance in Basil (Ocimum basilicum L.) and Parsley (Petroselinum crispum L.)
Source: Plants (Basel). 2021 Jul 7;10(7):1391. doi: 10.3390/plants10071391 (PMC8309453; doi:10.3390/plants10071391)

Supplementary material

Table S1. Complete compositions of the headspaces and essential oils of basil (*Ocimum basilicum* L.) leaves treated by foliar spraying with *Ulva intestinalis* extract, chitosan or water (control).

Compounds	l.r.i.[a]	Relative abundance (%)±SD[b]					
		Headspace			Essential oil		
		Control	Chitosan	*U. intestinalis* extract	Control	Chitosan	*U. intestinalis* extract
(*Z*)-3-hexen-1-ol	857	-[c]	-	-	tr[d]	-	-
α-pinene	941	0.95±0.09	1.68±0.06	1.07±0.13	-	-	-
1-octen-3-ol	980	-	-	-	tr	-	-
β-pinene	982	3.01±0.08^{B}	5.02±0.00^{A}	3.10±0.12^{B}	-	-	-
myrcene	993	1.65±0.03^{C}	4.15±0.13^{A}	2.50±0.22^{B}	tr	-	-
limonene	1032	-	-	-	tr	-	-
1,8-cineole	1034	18.63±0.02^{C}	44.74±0.16^{A}	26.15±0.12^{B}	3.38±0.01^{A}	1.23±0.21^{B}	0.63±0.05^{C}
(*E*)-β-ocimene	1052	7.99±0.14^{A}	-^{B}	7.91±0.02^{A}	0.13±0.00^{A}	-^{B}	0.13±0.02^{A}
cis-sabinene hydrate	1070	-	-	-	0.18±0.01	-	-
1-octanol	1071	-	-	-	tr	-	-
terpinolene	1088	1.24±0.06	2.34±0.00	0.60±0.04	tr	-	-

linalool	1101	2.61±0.03^{C}	8.32±0.04^{B}	11.87±0.04^{A}	10.57±0.13^{A}	2.20±0.31^{C}	3.35±0.57^{B}
allo-ocimene	1129	-	-	0.10±0.00	-	-	-
camphor	1143	0.49±0.04	1.33±0.00	0.33±0.02	0.31±0.00	-	-
borneol	1165	-	0.47±0.00	-	0.80±0.11	-	0.18±0.05
δ-terpineol	1167	tr	-	0.31±0.01	0.10±0.13	-	-
4-terpineol	1178	-	-	-	tr	-	-
α-terpineol	1189	0.47±0.01	0.76±0.00	0.69±0.01	1.17±0.03	-	0.29±0.07
geranial	1271	tr	-	-	-	-	-
bornyl acetate	1287	tr	-	-	0.74±0.01	2.67±0.02	0.90±0.19
methyl geranate	1325	-	0.11±0.00	-	-	-	-
δ-elemene	1340	0.49±0.03	0.73±0.01	0.34±0.01	-	-	-
α-cubebene	1350	tr	-	-	-	-	-
eugenol	1358	17.30±0.09^{A}	5.38±0.03^{C}	12.54±0.06^{B}	29.41±0.18^{A}	1.46±0.07^{C}	6.54±0.96^{B}
α-copaene	1376	0.24±0.01	0.31±0.00	0.22±0.01	tr	-	-
(*E*)-methyl cinnamate	1380	0.25±0.01	-	-	tr	-	-
β-cubebene	1390	-	-	-	tr	-	0.09±0.13
β-elemene	1392	1.63±0.02	2.38±0.01	1.91±0.02	0.09±0.13	0.15±0.21	0.35±0.06
methyl eugenol	1403	5.51±0.04^{A}	1.16±0.00^{B}	-^{C}	36.48±1.22^{A}	9.07±0.72^{C}	12.98±0.38^{B}
cis-α-bergamotene	1416	0.16±0.01	-	0.20±0.02	tr	-	-

β-caryophyllene	1420	0.87±0.01	0.97±0.00	0.67±0.01	0.22±0.01	0.42±0.09	0.71±0.11
β-copaene	1429	0.28±0.01	0.26±0.00	0.25±0.01	-	-	-
trans-α-bergamotene	1438	11.90±0.06[B]	0.72±0.01[C]	15.08±0.08[A]	4.69±0.06[C]	29.11±0.11[A]	14.40±0.59[B]
α-guaiene	1439	0.33±0.01	1.46±0.01	0.75±0.13	0.22±0.02	1.13±0.00	1.01±0.06
(*Z*)-β-farnesene	1444	0.22±0.02	-	0.23±0.00	tr	-	0.16±0.01
cis-muurola-3,5-diene	1447	0.14±0.01	0.31±0.01	0.19±0.01	tr	-	0.21±0.00
α-humulene	1456	1.94±0.03[A]	1.96±0.01[A]	0.88±0.01[B]	1.00±0.04[C]	4.06±0.04[A]	3.18±0.12[B]
(*E*)-β-farnesene	1460	14.03±0.07[A]	8.11±0.06[B]	4.30±0.00[C]	2.60±0.24[C]	8.44±0.07[A]	7.45±0.36[B]
cis-muurola-4(14),5-diene	1462	0.19±0.03	0.52±0.01	0.46±0.05	0.07±0.10	0.41±0.01	0.48±0.03
γ-muurolene	1477	tr	-	-	-	-	-
germacrene D	1478	2.17±0.00[A]	1.95±0.03[B]	1.74±0.01[C]	2.14±0.13[C]	5.47±0.40[B]	7.20±0.08[A]
β-selinene	1485	0.82±0.01	-	-	0.35±0.01	-	1.14±0
bicyclosesquiphellandrene	1489	tr	-	-	-	-	-
α-selinene	1494	-	-	-	tr	-	-
bicyclogermacrene	1496	0.86±0.00	0.79±0.04	0.49±0.02	0.67±0.03	3.28±0.07	2.79±0.12
α-muurolene	1498	-	0.12±0.16	-	-	-	-
α-bulnesene	1505	0.82±0.01	1.42±0.01	1.24±0.00	0.93±0.04	4.95±0.13	-
germacrene A	1506	-	-	-	-[B]	-[B]	5.41±0.21[A]
β-bisabolene	1509	0.41±0.00	-	0.30±0.00	tr	-	0.22±0.01

trans-γ-cadinene	1513	1.04±0.00^{C}	1.80±0.01^{A}	1.65±0.03^{B}	0.89±0.04^{C}	4.79±0.00^{A}	3.63±0.07^{B}
7-*epi*-α-selinene	1517	-	-	-	tr	-	-
trans-calamenene	1524	-	0.21±0.00	-	-	-	-
β-sesquiphellandrene	1525	0.80±0.01	-	0.72±0.04	0.17±0.03	0.69±0.13	0.96±0.09
cadina-1,4-diene	1534	-	-	-	-	0.15±0.21	-
cis-sesquisabinene hydrate	1545	-	-	-	tr	-	0.16±0.04
(*E*)-nerolidol	1565	-	-	-	-	-	0.18±0.03
germacrene D-4-ol	1575	-	-	-	-	-	0.06±0.08
caryophyllene oxide	1581	-	-	-	-	-	0.08±0.11
1-tridecanol	1582	-	0.12±0.01	-	-	-	-
1,10-*di-epi*-cubenol	1614	tr	-	-	0.18±0.02	1.03±0.02	1.59±0.12
epi-α-cadinol	1640	0.22±0.02^{B}	0.15±0.00^{C}	0.33±0.02^{A}	2.2±0.23^{B}	16.56±0.71^{A}	16.22±2.79^{A}
β-eudesmol	1650	-	-	-	0.18±0.04	0.62±0.12	1.39±0.23
α-cadinol	1654	-	-	-	-	0.50±0.01	0.85±0.11
*neo*intermedeol	1660	-	-	-	-	-	0.36±0.04
α-bisabolol	1683	-	-	-	-	-	0.35±0.01
methyl *p*-methoxycinnamate	1692	0.06±0.08	-	-	-	-	-
(*E,E*)-farnesyl acetate	1843	-	-	-	-	-	0.36±0.06
phytol	2114	-	-	-	-	-	3.03±0.76

Monoterpene hydrocarbons	14.83±0.09^B	13.18±0.20^C	15.26±0.10^A	0.13±0.00^A	-^B	0.13±0.02^A
Oxygenated monoterpenes	22.25±0.13^C	55.73±0.20^A	39.33±0.11^B	17.24±0.21^A	6.10±0.54^B	5.34±0.93^B
Sesquiterpene hydrocarbons	39.29±0.11^A	24.01±0.04^C	31.59±0.04^B	14.02±0.88^C	63.04±0.49^A	49.43±1.36^B
Oxygenated sesquiterpenes	0.22±0.02^B	0.15±0.00^C	0.33±0.02^A	2.55±0.30^B	18.70±0.60^A	21.57±3.08^A
Oxygenated diterpenes	-	-	-	-^B	-^B	3.03±0.76^A
Phenylpropanoids	23.11±0.04^A	6.54±0.03^C	12.54±0.06^B	65.89±1.41^A	10.53±0.79^C	19.52±1.34^B
Non-terpene derivatives	-^B	0.12±0.01^A	-^B	-	-	-
Total identified (%):	99.69±0.09	99.72±0.01	99.04±0.02	99.82±0.01	98.36±0.25	98.92±0.31

[a] Linear retention indices on a HP5-MS capillary column; [b] Standard deviation; [c] Not detected; [d] Traces, <0.1%. For the most abundant compounds and chemical classes of the same kind of sample (EO or headspace), along the same row, different superscript uppercase letters (A,B,C) indicate significant differences (Tukey's HSD, P < 0.05) among the samples.

Table S2. Complete compositions of the headspaces and essential oils of parsley (*Petroselinum crispum* L.) leaves treated by foliar spraying with UIE, chitosan or water (control).

Compounds	l.r.i.[a]	Relative abundance (%)±SD[b]					
		Headspace			Essential oil		
		Control	Chitosan	*U. intestinalis* extract	Control	Chitosan	*U. intestinalis* extract
α-pinene	941	1.82±0.00	0.67±0.00	0.69±0.01	-[c]	0.13±0.01	tr
sabinene	976	-	-	-	-	-	tr
β-pinene	982	0.38±0.01	tr[d]	tr	-	tr	tr
3-octanol	993	-	-	-	-	-	tr
myrcene	993	1.74±0.20	2.66±0.23	1.41±0.10	0.19±0.02	1.37±0.01	0.96±0.13
α-phellandrene	1005	1.42±0.25	1.09±0.25	1.19±0.00	tr	0.40±0.00	0.29±0.03
p-cymene	1027	-	-	-	-	-	tr
β-phellandrene	1031	28.09±0.17[A]	14.57±0.05[C]	15.23±0.02[B]	1.04±0.03[C]	7.38±0.01[A]	5.42±0.44[B]
(*E*)-β-ocimene	1052	-	-	-	-	-	tr
γ-terpinene	1062	0.67±0.01	0.32±0.00	0.12±0.16	-	0.16±0.00	0.11±0.01
cis-sabinene hydrate	1070	-	-	-	-	-	tr
terpinolene	1088	3.63±0.01	1.65±0.02	3.20±0.01	0.22±0.01	0.75±0.01	0.86±0.10
linalool	1101	-	-	-	-	tr	tr
nonanal	1102	-	-	-	-	-	tr

1,3,8-*p*-menthatriene	1113	17.82±0.06[C]	26.87±0.02[B]	40.8±0.16[A]	2.95±0.08[C]	11.04±0.01[A]	10.26±0.63[B]
α-terpineol	1189	-	-	0.09±0.11	-	-	tr
cis-dihydrocarveol	1195	-	-	-	-	-	tr
decanal	1204	tr	-	-	-	-	tr
β-cyclocitral	1217	-	0.45±0.03	-	-	-	-
citronellol	1230	-	-	-	-	-	tr
n-tridecane	1300	0.07±0.08	tr	tr	-	-	-
δ-elemene	1340	-	tr	-	-	-	-
α-terpinyl acetate	1352	tr	-	0.15±0.00	tr	-	tr
3-methyl tridecane	1371	-	0.14±0.01	-	-	-	-
α-copaene	1376	0.67±0.00	0.93±0.01	1.27±0.01	-	-	tr
β-bourbonene	1384	-	tr	tr	-	-	-
β-cubebene	1390	0.23±0.00	0.41±0.00	0.51±0.01	-	-	-
1-tetradecene	1392	0.15±0.00	0.19±0.00	-	-	-	-
β-elemene	1392	-	-	0.22±0.00	-	-	-
n-tetradecane	1400	0.41±0.00	0.37±0.00	0.32±0.01	-	-	-
β-caryophyllene	1420	6.21±0.03[B]	7.01±0.01[A]	5.45±0.02[C]	0.95±0.04[B]	0.40±0.01[C]	1.98±0.22[A]
β-copaene	1429	0.28±0.00	0.67±0.02	0.64±0.00	-	-	-
γ-elemene	1433	tr	-	0.65±0.02	-	-	-

trans-α-bergamotene	1438	-	tr	tr	-	-	tr
aromadendrene	1441	-	0.16±0.00	0.14±0.00	-	-	-
α-humulene	1456	0.22±0.00	0.57±0.01	0.52±0.01	0.07±0.08	-	0.06±0.07
(*E*)-β-farnesene	1460	tr	0.11±0.01	0.10±0.00	-	-	0.16±0.04
cis-muurola-4(14),5-diene	1462	-	0.11±0.01	0.11±0.00	-	-	-
germacrene D	1478	0.68±0.02^{C}	3.57±0.01^{A}	3.31±0.01^{B}	0.55±0.01^{B}	0.29±0.01^{C}	1.6±0.21^{A}
n-pentadecene	1488	0.08±0.11	-	0.23±0.00	-	-	-
valencene	1492	-	0.24±0.00	-	-	-	-
bicyclogermacrene	1495	tr	-	0.19±0.00	-	-	tr
β-dihydro agarofuran	1498	-	0.46±0.01	0.1±0.00	0.05±0.07	-	0.14±0.00
n-pentadecane	1500	0.76±0.03	0.55±0.02	0.46±0.04	tr	-	tr
β-bisabolene	1509	0.16±0.23	0.24±0.00	0.21±0.00	0.11±0.14	-	0.47±0.07
myristicin	1522	2.49±0.01^{C}	9.46±0.11^{B}	14.23±0.06^{A}	10.66±0.18^{A}	4.15±0.04^{C}	8.80±0.35^{B}
β-sesquiphellandrene	1524	4.66±0.01^{A}	2.94±0.16^{B}	1.52±0.04^{C}	6.34±0.23^{B}	2.34±0.02^{C}	6.91±0.09^{A}
kessane	1530	-	0.37±0.00	-	0.11±0.16	-	0.16±0.02
elemol	1549	-	-	-	-	-	0.10±0.14
elemicin	1556	tr	-	0.13±0.18	-	tr	0.39±0.06
ledol	1566	-	1.64±0.03	-	-	-	-
3-methyl pentadecane	1570	0.22±0.30	-	-	-	-	-

carotol	1594	-	-	tr	-	-	0.05±0.07
n-hexadecane	1600	tr	-	-	-	-	-
epi-α-cadinol	1640	-	0.14±0.03	tr	-	-	-
apiole	1684	26.66±0.11^{A}	20.41±0.02^{B}	4.85±0.01^{C}	76.79±0.85^{A}	71.64±0.01^{B}	61.27±2.64^{C}
Monoterpene hydrocarbons		55.56±0.19^{B}	47.81±0.11^{C}	62.63±0.13^{A}	4.4±0.02^{C}	21.21±0.01^{A}	17.9±1.34^{B}
Oxygenated monoterpenes		-^{B}	-^{B}	0.23±0.11^{A}	-	-	-
Sesquiterpene hydrocarbons		13.11±0.29^{C}	16.93±0.14^{A}	14.82±0.13^{B}	8.00±0.42^{B}	3.02±0.02^{C}	11.16±0.71^{A}
Oxygenated sesquiterpenes		-^{C}	2.61±0.01^{A}	0.1±0.00^{B}	0.16±0.23^{A,B}	-^{B}	0.45±0.23^{A}
Apocarotenoids		-^{B}	0.45±0.03^{A}	-^{B}	-	-	-
Phenylpropanoids		29.14±0.11^{B}	29.86±0.13^{A}	19.21±0.11^{C}	87.45±0.66^{A}	75.78±0.03^{B}	70.45±2.35^{C}
Non-terpene derivatives		1.68±0.53^{A}	1.24±0.03^{A,B}	1.00±0.04^{B}	-	-	-
Total identified (%):		99.48±0.74	98.90±0.05	97.98±0.08	100±0.00	100±0.00	99.95±0.08

[a] Linear retention indices on a HP5-MS capillary column; [b] Standard deviation; [c] Not detected; [d] Traces, <0.1%. For the most abundant compounds and chemical classes of the same kind of sample (EO or headspace), along the same row, different superscript uppercase letters (A,B,C) indicate significant differences (Tukey's HSD, $P < 0.05$) among the samples.

Figure S1. Elution profile of U. intestinalis extract (UIE) as determined by high-pressure size-exclusion chromatography (HPSEC) coupled to refractive index (RI), and multi-angle laser light scattering (MALLS) detectors.

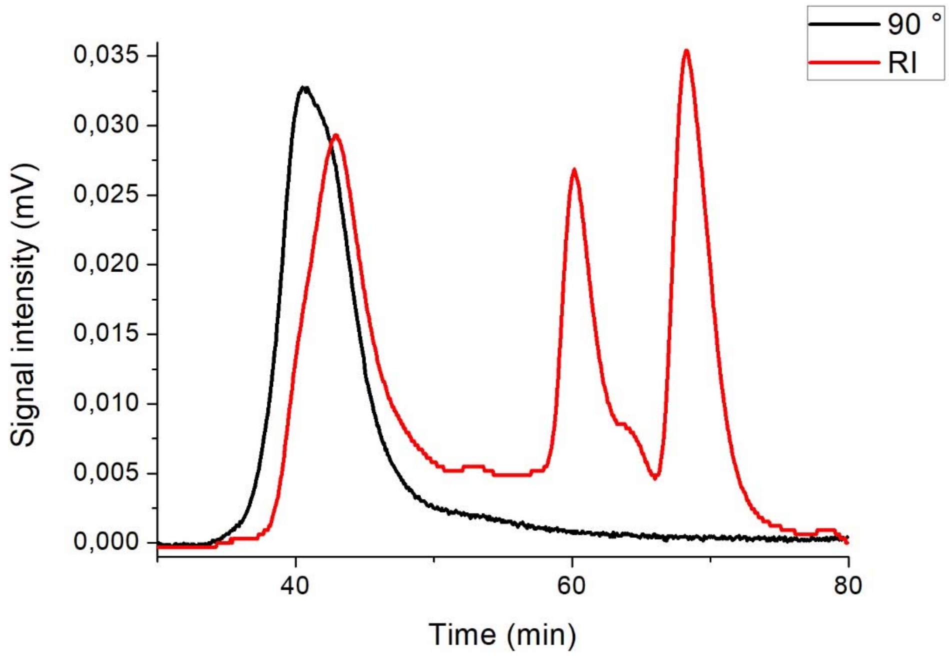

Figure S2. Loadings plot of the principal component analysis (PCA) performed on the complete compositions of basil leaf headspaces (HS) and essential oils (EO).

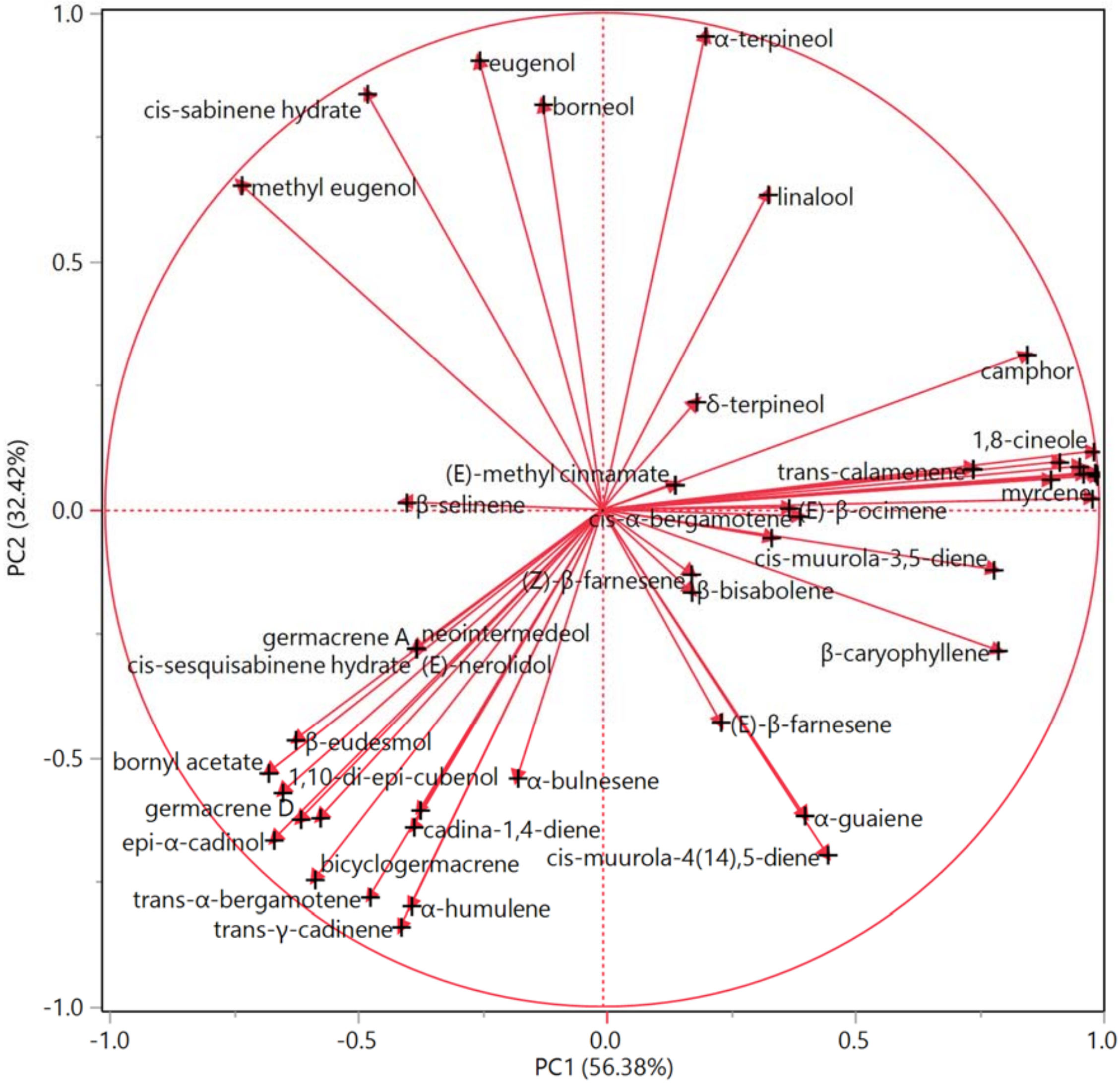

Figure S3. Loadings plot of the principal component analysis (PCA) performed on the complete compositions of parsley leaf headspaces (HS) and essential oils (EO).

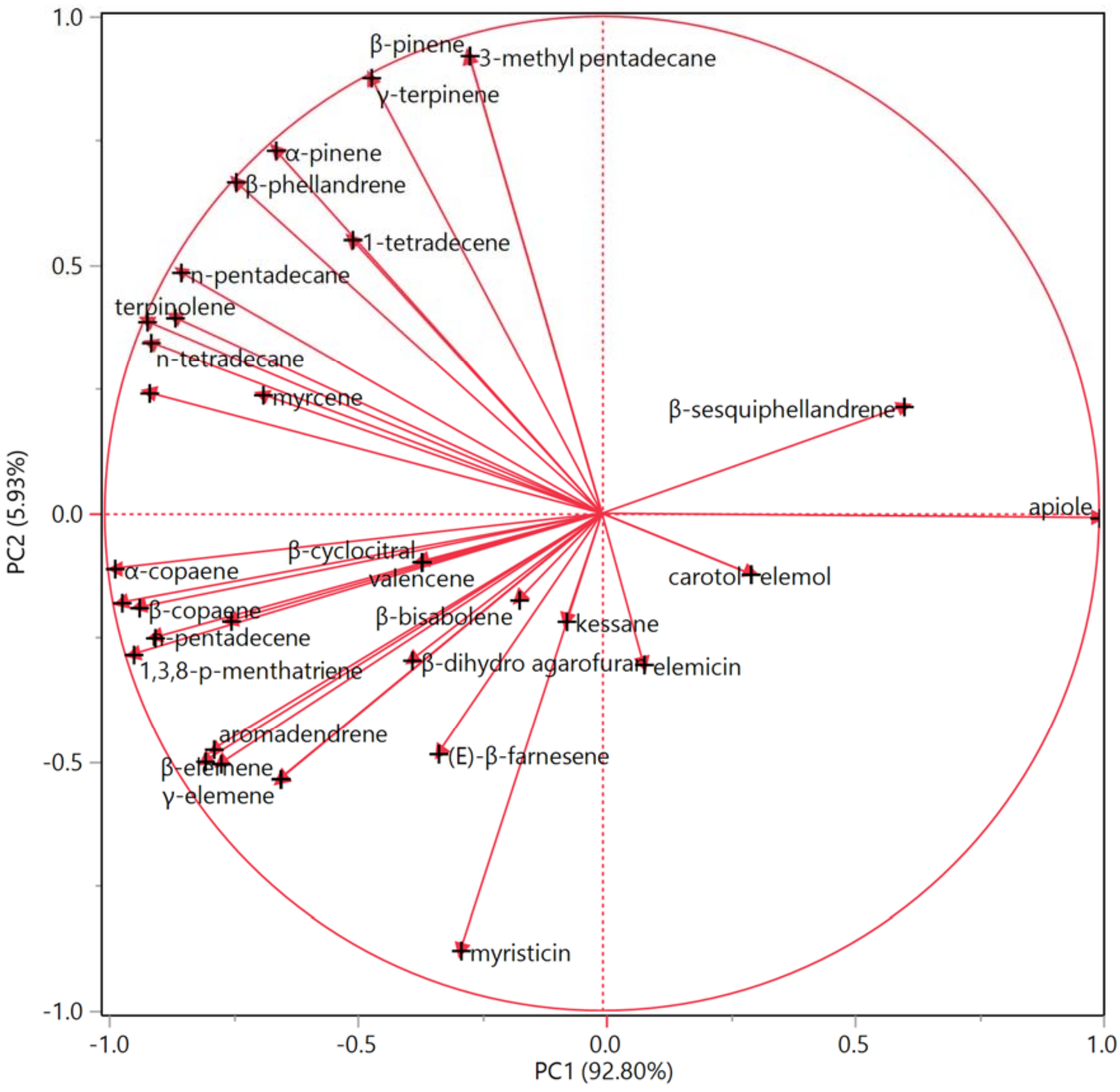

Supplement: Supplementary file 1 [file plants-10-01391-s001.zip › plants-1258404-supplementary.pdf]
